# Metal-organic frameworks/metal nanoparticles as smart nanosensing interfaces for electrochemical sensors applications: a mini-review

**DOI:** 10.3389/fbioe.2023.1251713

**Published:** 2023-08-08

**Authors:** Min Jiang, Jing Liao, Chenghao Liu, Jun Liu, Peixian Chen, Jia Zhou, Zhizhi Du, Yan Liu, Yan Luo, Yangbin Liu, Fei Chen, Xiaojun Fang, Xiaofeng Lin

**Affiliations:** ^1^ Key Laboratory of Prevention and Treatment of Cardiovascular and Cerebrovascular Diseases of Ministry of Education, Key Laboratory of Biomaterials and Biofabrication in Tissue Engineering of Jiangxi Province, Key Laboratory of Biomedical Sensors of Ganzhou, School of Medical and Information Engineering, School of Pharmacy, Scientific Research Center, Gannan Medical University, Ganzhou, China; ^2^ Department of Neurosurgery, The Second Affifiliated Hospital of Nanchang University, Nanchang, China; ^3^ Department of Neurosurgery, The Affiliated Ganzhou Hospital of Nanchang University, Ganzhou, China; ^4^ Department of Health Services, Fujian Hwa Nan Women’s College, Fuzhou, China

**Keywords:** metal-organic frameworks, metal nanoparticles, composite, smart nanosensing, electrochemical sensor

## Abstract

Metal-organic frameworks (MOFs) are porous materials with huge specific surface area and abundant active sites, which are composed of metal ions or clusters and organic ligands in the form of coordination bonds. In recent years, MOFs have been successfully applied in many fields due to their excellent physical, chemical, and biological properties. Electrochemical sensors have advantages such as economy, portability, and sensitivity, making them increasingly valued in the field of sensors. Many studies have shown that the electrode materials will affect the performance of electrochemical sensors. Therefore, the research on electrode materials is still one of the hotspots. MOFs are also commonly used to construct electrochemical sensors. However, electrochemical sensors prepared from single MOFs have shortcomings such as insufficient conductivity, low sensitivity, and poor electrochemical catalytic ability. In order to compensate for these defects, a new type of nanocomposite material with very ideal conductivity was formed by adding metal nanoparticles (MNPs) to MOFs. The combination of the two is expected to be widely applied in the field of sensors. This review summarizes the applications of various MNPs/MOFs composites in the field of electrochemical sensors and provides some references for the development of MNPs/MOFs composites-based electrochemical sensors in the future.

## 1 Introduction

Coordination polymers are framework materials with a periodic spatial network structure, consisting of metal ions (or metal clusters) assembled with multi-dentate ligands through coordination bonds (Liu J.-Q. et al., 2020; Daniel et al., 2022). Metal-organic frameworks (MOFs), a subset of coordination polymers, are a class of coordination networks with both organic ligands and potential pores (Falsafi et al., 2021; Kajal et al., 2022). In comparison with other inorganic porous materials, such as zeolites, porous silica, and carbon materials, MOFs possess some unique structural advantages, including structural diversity, huge specific surface area, and high porosity, abundant unsaturated metal sites and good biocompatibility (Liu C.-S. et al., 2020; Çorman et al., 2022; Zhang W. et al., 2023). Based on these attractive structural and performance advantages, MOFs have attracted many researchers to investigate the structural features and functions of MOFs and explore their potential applications in gas adsorption, multiphase catalysis, drug delivery, biomedical imaging, and chemical sensing (Lu et al., 2018; Lin et al., 2020; Bag et al., 2021; Kumari et al., 2022).

The electrochemical sensor is a kind of special analysis and detection platform, which applies electrochemical detection technology to the sensor. It consists of three parts: an identification probe, a power converter, and a data analyzer. It works by first modifying the identification materials on the electrode surface by chemical or physical methods. And then the target molecules on the surface of the electrode are captured through intermolecular-specific recognition. Finally, their concentration signals are converted into recognizable electrical signals, such as current, voltage and impedance, etc. Due to their simple operation, economy, convenient detection, high sensitivity, and fast response, electrochemical biosensors have been widely used in biomedicine, food engineering, environmental monitoring, and other fields (Chen et al., 2018; Huang et al., 2020; Lin et al., 2021a; Lin et al., 2021b; Mei et al., 2022a; Mei et al., 2022b; Huang et al., 2022). It is well known that the sensor interface materials of electrochemical sensors play an important role in the efficient and sensitive detection of different target molecules. Accordingly, the reasonable choice of sensing interface materials will determine the performance of electrochemical sensors.

At present, some MOFs meet the requirements of electrochemical sensing that can be directly used to construct sensing interfaces. For example, MOFs with electroactivity can be used as electrocatalysts to detect small molecule compounds such as H_2_O_2_(Li et al., 2022), glucose (Adeel et al., 2021), ascorbic acid (Wang H. et al., 2021), nitrite (Yang et al., 2022), dopamine (Ma J. et al., 2021), etc. Nevertheless, MOFs have some drawbacks that cannot be ignored. For instance, their physical and chemical stability tends to be poorer than that of conventional inorganic porous materials, which limits the application scope of MOFs to a certain extent. As a result, the frameworks of MOFs have been functionally modified to form composites to obtain specific functions and make up for some shortcomings of MOFs. For the practical application of MOFs-modified electrodes, the performance of MOFs can be improved in two ways by improving the electrical conductivity of MOF-based materials and designing MOF-based materials with redox activity (Zhou et al., 2022). The porous nature of MOFs enables them to be integrated with a variety of functional materials such as metal nanoparticles, carbon nanomaterials, polymers, biomolecules, etc., to form composites that combine the advantages of MOFs with other active materials and exhibit superior electrocatalytic/electrochemical sensing properties than MOFs alone.

Metal nanoparticles (MNPs) have become a hot topic of current research (Mourdikoudis et al., 2018; Yi et al., 2022; Zhang et al., 2022; Zhang Z. et al., 2023). MNPs have outstanding optical and conductive properties (Wang et al., 2020), which have been extensively applied in the sensing field in recent years. The large surface area and ordered porous structure of MOFs enable functionalized metal nanoparticles (MNPs) to be anchored on the surface of MOFs or encapsulated in cavities/pores to form MNPs/MOFs composites (Liu C.-S. et al., 2020). The combination of large specific surface area and size-limiting effect of MOFs and catalytic activity and conductivity of MNPs provides more catalytic active sites and a good microenvironment, making MNPs/MOFs composites excellent in both catalytic and sensing properties (Wu and Zhao, 2017; Jiao et al., 2018; Zhong et al., 2019; Zhang W. et al., 2020; Hao et al., 2021; Zhang et al., 2021; Guo M. et al., 2022; Guo T. et al., 2022; Xu et al., 2023). Here, this review summarizes the relevant literature that has been reported on the preparation of MNPs/MOFs composites for electrochemical sensors (Table 1).

**TABLE 1:** Electrochemical sensors based on MNPs/MOFs composites.

Materials	Targets	Electrochemical methods	Linear range	Detection limit	Ref
PtNi@Cu-TCPP(Fe)	calprotectin	CV, EIS	200 fg/mL-50 ng/mL	137.7 fg/mL	Dong et al. (2020)
AuNPs/Co-MOF/MWCNT	nitrite	CV, EIS	1-1,000 μmol/L	0.4 μmol/L	Lei et al. (2021)
CoNi-MOF	miRNA-126	EIS	1.0 fmol/L-10.0 nmol/L	0.14 fmol/L	Hu et al. (2021)
Ce-Zn-MOF/MWCNT	bisphenol A	CV, EIS, DPV	0.1–100 μmol/L	7.2 nmol/L	Huang et al. (2021a)
Co-MOFs	glucose	CV	10–1,200 μmol/L	3.2 μmol/L	Ma et al. (2021b)
Au NPs/UiO-66	H_2_O_2_	CV, EIS	0.2–23 mmol/L	0.045 μmol/L	Wang et al. (2021b)
Pd@UiO-66	microRNA-21	DPV	20 fmol/L-600 pmol/L	0.713 fmol/L	Meng et al. (2020a)
Ag@MOF	Cu(II)	CV	—	0.68 μmol/L	Kwon and Kim (2021)
Ag@MOF	Pb(II)	CV	—	0.64 μmol/L	Kwon and Kim (2021)
USAuNPs@AuZn-MOF	estrone	CV, EIS, DPV	0.05 µmol/L-5 µmol/L	12.3 nmol/L	Chai et al. (2021)
Ag-ZIF-67p	Acetaminophen	CV, DPV	—	0.2 μmol/L	Tang et al. (2020)
Ag-ZIF-67p	dopamine	CV, DPV	—	0.05 μmol/L	Tang et al. (2020)
Ag-CoNi-MOF	luteolin	CV, DPV	0.002–1.0 μmol/L	0.4 nmol/L	Tang et al. (2022)
Co_x_Ni_3-x_ (HITP)_2_	enrofloxacin	CV, EIS	0.001–1 pg/mL	0.2 fg/mL	Song et al. (2021)
Cu@C@ZIF-8	nitrite	CV, EIS, DPV	0.1–300.0 μmol/L	0.033 μmol/L	Gao et al. (2022)
CoP@C/NCS/GCE	dopamine	SWV	5.0–400.0 μmol/L	0.03 μmol/L	Xiao et al. (2021)
NiCu-MOF-6	glucose	CV	0.02-4.93 mmol/L	15 μmol/L	Pan et al. (2021)
Ni@Cu-MOF	glucose	CV	5–2,500 μmol/L	1.67 μmol/L	Xue et al. (2020)
Au@ZIF-8	dopamine	CV, DPV	0.1–50 μmol/L	0.01 μmol/L	Lu et al. (2020)
Ni-MOF	dopamine	DPV	0.2–100 μmol/L	60 nmol/L	Huang et al. (2021b)
CuCo-MOFs/NF	glucose	CV	—	0.23 mmol/L	Du et al. (2022)
Au/Co-BDC/MoS_2_	Cardiac troponin I	EIS	10 fg/mL–100 ng/mL	3.02 fg/mL	Zhao et al. (2021)
Au@MOFs	Neuron-specific enolase	DPV, EIS	10 fg/mL–100 ng/mL	4.17 fg/mL	Ma et al. (2020)
Au NPs@ZIF-8	carcinoembryonic antigen	DPV, EIS	5 pg/mL-400 ng/mL	1.3 pg/mL	Ye et al. (2020)
Au@UiO-66(NH_2_)	HBsAg	CV, DPV	1.13–fg/mL-100 ng/mL	1.13 fg/mL	Bajpai et al. (2021)
Au/UiO-66-NH_2_	streptomycin	CV, DPV	0.005-150 ng/mL	2.6 pg/mL	Meng et al. (2020b)
MIP/Pt-UiO-66/CPME	phosalone	CV, DPV	0.50–nmol/L-20 μmol/L	0.078 nmol/L	Xu et al. (2020)
AgNPs/PCN-224	telomerase	CV, EIS, DPV	1.0 × 10^-7^-1.0 × 10^−1^ IU/L	5.4 × 10^−8^ IU/L	Wang et al. (2021c)
Fe-Cu-BTC	bisphenol A	CV, DPV	0.1-1.0 μM	18 nM	Nguyen et al. (2022)
Fe-rich FeCoNi-MOF	imidaclopri	CV, DPV	1 pmol/L–120 μmol/L	0.04 pmol/L	Shu et al. (2023)
Mn_3_O_4_@ZIF-67	glucose	Amperometric method	0.0008–6.0 mM	0.24 μM	Dong et al. (2022)
Fe@YAU-101	Cd^2+^	CV, EIS, DPV	0.003–42 μM	1 nM	Liang et al. (2023)
Fe@YAU-101	Pb^2+^	CV, EIS, DPV	0.004–80 μM	1.33 nM	Liang et al. (2023)
Fe@YAU-101	Hg^2+^	CV, EIS, DPV	0.045–66 μM	15 nM	Liang et al. (2023)
Au-CH@MOF-5	topotecan (TPT)	CV, EIS, DPV	0.4–70.0 nM	0.298 nM	Mehmandoust et al. (2023)
NiMn-LDH-MOF	glucose	CV	4.9 μM–2.2 mM	0.87 μM	Wei et al. (2023)
Ni/Co-FAMOF	glucose	CV, EIS	0.006–1.004 mM	2 μM	Song et al. (2023)
Ag@MOF-199	Ni^2+^	CV	-	6.06 nM	Bodkhe et al. (2023)
AuNPs/Zr-MOF-Graphene	sunset yellow	CV, DPV	0.1–1,000 μM	0.1 μM	Sun et al. (2023)
AuNPs/Zr-MOF-Graphene	Sudan I	CV, DPV	0.1–800 μM	0.1 μM	Sun et al. (2023)
AuNP/Cu-TCPP(Fe)	lactate (LA)	CV, EIS	0.013 nM-100 mM	0.91 p.m.	Ji et al. (2023)

This paper has reviewed the research progress of electrochemical sensors based on MNPs/MOFs in recent years. Meanwhile, the future challenges and opportunities for the preparation of electrochemical sensors based on MNPs/MOFs are briefly discussed. In conclusion, we aim to provide some new ideas for opening up MNPs/MOFs’ high-performance electrochemical sensors.

## 2 MOFs/MNPs-based electrochemical sensors

In recent years, MOFs are often used as a carrier to encapsulate MNPs, which not only effectively prevent MNPs agglomeration but also have the following functions: 1) as a protective layer, they effectively prevent the aggregation and migration of MNPs. 2) Their inherent large surface area and porosity can facilitate the migration of reactants on the MNPs’ surface. 3) Maintaining the catalytic activity of MNPs in multiple catalytic cycles. Compared with pure MOFs, MNPs@MOFs composites with core-shell heterogeneous structures have better catalytic and adsorption properties, and are beneficial for improving selectivity due to their size-screening effect. There are three general methods for the synthesis of MNPs@MOFs composites (Li et al., 2018). The first method is to confine the MNPs to the cavity or pore of the MOFs in the form of a ship-in-a-bottle, and the common methods include impregnation, coprecipitation, and deposition-precipitation. Unfortunately, some precursors may be deposited on the outer surface of MOFs to form aggregates, making them difficult to precisely control the MNPs at the loading position of MOFs. The second and third methods are more commonly used to encapsulate the MNPs in a bottle-around-ship form by MOFs. In the second method, MNPs with a well-defined structure are first synthesized and then mixed with the precursor solution of MOFs to obtain an encapsulated structure. Thus, the most important point to obtain a well-defined structure is to avoid agglomeration of MNPs and self-core of MOFs, The third method, in which the MNPs are embedded in the MOFs framework by one-pot synthesis, is considered the preferred method due to their low production cost/time and ease of scale-up. Commonly used metal nanoparticles in electrochemical sensors are gold nanoparticles, silver nanoparticles, copper nanoparticles, nickel nanoparticles, etc.

### 2.1 MOFs/Au NPs-based electrochemical sensors

**FIGURE 1:**
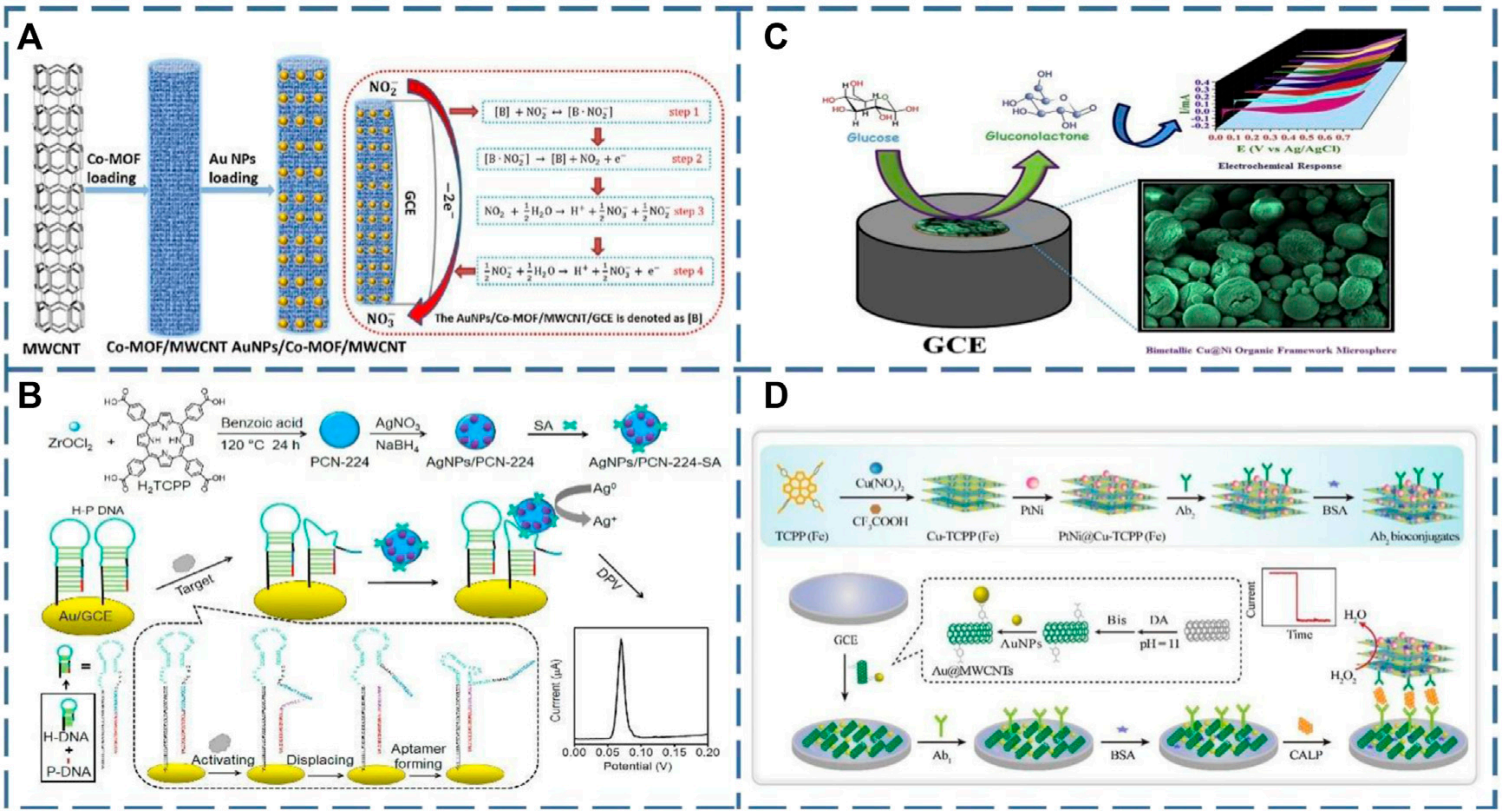
**(A)** Schematic illustration of the synthesizing process for the AuNPs/Co-MOF/MWCNT and the sensing mechanism of nitrite (Lei et al., 2021); **(B)** A schematic diagram of the electrochemical sensing strategy of telomerase activity based on the construction of streptavidin (SA) modified AgNPs/PCN-224 (Dong et al., 2019); **(C)** Pictorial representation of bimetallic Cu@Ni organic framework for electrochemical glucose oxidation (Kim and Muthurasu, 2020); **(D)** Schematic illustration of the construction process of the sandwich electrochemical immunosensor (Dong et al., 2020).

To the best of our knowledge, there have been a large number of literature reports on the application of Au@MOFs in electrochemical sensors for the detection of different targets (Chen et al., 2019; Meng X. et al., 2020; Bajpai et al., 2021; Lei et al., 2021). At the same time, the introduction of carbon materials can efficiently improve the conductivity of pristine MOFs and limit their growth and aggregation. Consequently, the hybridization of MNPs@MOFs with carbon materials will also produce sensors with excellent stability and conductivity and give MNPs@MOFs template effect. For example, Zhang and his colleagues used Au nanoparticles to modify ZIF-8 as a basis for building a sensing platform to detect carcinoembryonic antigens (CEA). The Au@ZIF-8 that was successfully synthesized helped to increase the loading of the antibody. Ordered mesoporous carbon, a novel carbon nanomaterial with ordered pores, which was then attached to the electrode surface to enhance the conductivity of this sensor (Zhang Y. et al., 2020). Furthermore, Lei and his teams prepared ternary composites consisting of multi-walled carbon nanotubes (MWCNT) as a substrate, cobalt-based metal-organic backbone (Co-MOF), and gold nanoparticles (AuNPs/Co-MOF/MWCNT) (Figure 1A), which exhibited efficient catalytic activity and highly sensitive response to nitrite (Lei et al., 2021). Small Co-MOF nanoplates were first grown *in situ* on the surface of conducting multi-walled carbon nanotubes, which could absorb a large amount of Au^+^. The reduced AuNPs can be uniformly restricted to Co-MOF/MWCNT. On the one hand, the binding of multi-walled carbon nanotubes to Co-MOF can markedly enhance the electron transport capacity of Co-MOF. On the other hand, the AuNPs distributed on Co-MOF can reduce the working voltage and significantly improve its catalytic activity for nitrite oxidation.

### 2.2 MOFs/Ag NPs-based electrochemical sensors

In addition to Au nanoparticles, Ag nanoparticles are also suitable as classical electrochemical detection labels. For instance, Wang and his colleagues took advantage of the fact that Ag nanoparticles are not easily oxidized and modified them on a porphyrin Zr-MOF (PCN-224) surface (AgNPs/PCN-224), functionalizing the surface with a streptavidin (SA) recognition element (Wang Y. et al., 2021). A hairpin structure of DNA is then modified on the electrode, and the developed sensor can be used to detect telomerase activity in cancer cells (Figure 1B). Zheng and his co-workers were also successful in embedding AgNPs into the ZIF-67 framework to form an ideal Ag@ZIF-67 core/shell material. The porous structure and large surface area of ZIF-67 provide abundant active sites for H_2_O_2_ adsorption and reduction as well as fast H_2_O_2_ diffusion channels (Dong et al., 2019). Moreover, the excellent electrical conductivity of AgNPs accelerates the electron transfer capability during H_2_O_2_ reduction, resulting in a highly sensitive electrochemical H_2_O_2_ sensor.

### 2.3 MOFs/Pt NPs-based electrochemical sensors

Pt nanoparticles, as noble metal nanoparticles, also have salient electrical conductivity and electrocatalytic activity and are among the superior materials for enhancing the conductivity of MOF-based sensors. Ma and his colleagues reported the synthesis of nanocomposite Pt@UiO-66-NH_2_ by immobilizing Pt NPs on the surface of UiO-66-NH_2_ and applying it to the preparation of acetylcholinesterase (AChE) biosensors (Ma et al., 2019). The pores of UiO-66-NH_2_ can restrict the aggregation of Pt NPs and help the transfer of Pt NPs. At the same time, it can also provide more AChE active sites. Thus, the sensor can achieve sensitive detection of organophosphorus pesticides (OPs). Deng and his colleagues reported a one-pot hydrothermal reduction of metals with three different ligand lengths of Zr-MOF(Deng et al., 2018). And TEM results demonstrated that the Pt@UiO-66 composites maintained the integrity of the UiO-66 framework with some Pt NPs dispersed within the framework. Meanwhile, aggregated Pt NPs were observed in the Pt@UiO-68 composites, which could be attributed to the exudation and aggregation of Pt nanoparticles due to the large pore size of UiO-68. However, the doping of Pt NPs led to the collapse of the UiO-67 crystal structure. The framework structure of Pt@Zr-MOF has a great influence on the electrocatalytic activity and induction of N_2_H_4_, where Pt@ UiO-66 has the best performance, followed by Pt@UiO-68, and Pt@UiO-67 has the worst electrocatalytic performance.

### 2.4 MOFs/Ru NPs-based electrochemical sensors

Ruthenium belongs to the platinum group of noble metals along with platinum. Although ruthenium nanoparticles have the advantages of electrochemical catalysis, charge storage, and electrode modification, they usually suffer from the defects such as poor porosity, poor stability, and low electron transfer rate (Abdelwahab et al., 2020). Therefore, bimetallic nanoparticles, especially bimetallic Pt and Ru nanoparticles possess more attractive electrocatalytic properties, large specific surface area, good stability, and higher sensitivity than single Ru nanoparticles (Lavanya et al., 2020). Cao and his colleagues prepared nanocomposites of MOFs encapsulating bimetallic nanoparticles (PtRu/UiO66-NH_2_) by embedding PtRu bimetallic nanoparticles (PtRu NPs) in UiO66-NH_2_(Cao et al., 2020). Subsequently, PtRu/UiO66-NH_2_ was subjected to high-temperature carbonization to synthesize PtRu-PC for the detection of uranium in aqueous solution. Among them, UiO66-NH_2_ could not only limit the aggregation and migration of PtRu NPs, but also improve the electrical conductivity. Raju et al. (2023) also synthesized a novel nanocomposite of ZIF-67-modified bimetallic platinum and ruthenium nanoparticles (Pt-Ru@C/ZIF-67) and constructed a biosensor based on this composite for the detection of saxitoxin (STX)). ZIFs are a class of MOFs consisting of imidazolyl ligands and transition metal cations, which have the advantages of high stability, large surface area, and tunable pore structure. The electrochemical conductivity of the Pt-Ru@C/ZIF-67 nanocomposites was improved by the synergistic interaction between bimetallic PtRu NPs and ZIF-67. In addition, the unique porous structure of the composite and the presence of Co easily adsorb STX adsorption, thus facilitating STX detection.

### 2.5 MOFs/Cu NPs-based electrochemical sensors

Although noble metal nanoparticles (such as Au, Ag, Pt, etc.) are excellent materials for building sensors, their scarcity and high cost are disadvantages that do not facilitate their dissemination. Hence, researchers started to explore the possibility of non-precious metal nanoparticles (e.g., Cu, Co, Ni, etc.). In 2016, Shi and his colleagues informed for the first time that their team utilized the *in situ* synthesis of Cu NPs inside the ZIF-8 cavity. The Cu NPs@ZIF-8 nanocomposite was successfully applied to construct a novel electrochemical glucose sensor (Shi et al., 2016). In addition, due to the low loading of MNPs on MOFs and slow electron migration during encapsulation, Kim and his colleagues synthesized a metal-organic bimetallic skeleton structure based on a Cu@Ni solid spherical structure using a two-step hydrothermal method to solve these problems. They then modified it on a glassy carbon electrode (GCE), which can be regarded as a non-enzymatic sensor for glucose detection in an alkaline solution (Figure 1C) (Kim and Muthurasu, 2020). The sensor displayed better electrocatalytic activity for glucose oxidation compared to the sensors prepared from individual material. Its excellent catalytic performance for glucose mainly depends on the synergistic effect of Cu and Ni MOF. It also effectively prevents the oxidation of common interfering biomolecules, including ascorbic acid, dopamine and uric acid. This bimetallic Cu@Ni organic backbone microsphere electrode has high reliability and accuracy and is suitable as an alternative electrode for non-enzymatic glucose sensors.

### 2.6 MOFs/other NPs-based electrochemical sensors

In addition to these common MOF-based electrochemical sensors constructed with metal nanoparticles, there are also bimetallic MOFs, which exhibit even more excellent electrochemical properties. Dong and his co-workers designed a novel composite with high electrocatalytic activity by functionalizing 2D ultra-thin Cu-TCPP(Fe) nanoflakes (PtNi@Cu-TCPP(Fe)) with PtNi nanospheres (Dong et al., 2020). The bimetallic Cu-TCPP(Fe) nanosheets have a huge specific surface area and a good deal of accessible active centers, allowing multiple PtNi to attach to their surfaces. Not only does it strengthen enzyme-free catalysis and conductivity, but it also provides junction sites for the immobilization of antibodies modified with fiducials. They constructed a sandwich-type calcium-binding protein immunosensor by exploiting the dual electrocatalytic activity of Cu-TCPP (Fe) and PtNi to amplify the signal for H_2_O_2_ reduction (Figure 1D). However, the immobilization of MNPs on the MOFs surface by simple sonication or surface self-assembly strategies will inevitably lead to the aggregation of some metal nanoparticles, which reduces the catalytic activity of MNPs. To further improve the analytical performance of electrochemical sensors, more effective active sites, and electrocatalysts are needed to be uniformly dispersed on the electrode surface. To achieve this goal, Chen and his colleagues used electrodeposition to uniformly disperse a large number of Au NPs (<200 nm) on the surface of Cu-MOF. Compared with the nitrite electrochemical sensor prepared by these two composites, the electrochemical sensor with electrodeposited Au/Cu-MOF has a wider linear detection range (Chen et al., 2019).

## 3 Conclusion and prospects

Within the last few years, the literature related to electrochemical sensors based on MNPs/MOFs composites has increased dramatically, and most of them focus mainly on the detection of glucose, hydrogen peroxide, and dopamine. The innovation of this mini-review is to systematically summarize the studies on electrochemical sensors related to MNPs/MOFs composites. The synthesis of MNPs/MOFs composites and the advantages of electrochemical sensors prepared using these composites are also briefly discussed. In addition, more detailed statistics of the studies on the detection of various analytes using the sensors are also presented. In addition, MNPs/MOFs composites utilize the synergistic effect between MNPs and MOFs: 1) MNPs act as active centers and MOFs play a stabilizing role. 2) MOFs limit the aggregation of MNPs. However, the sensor also has some drawbacks. First, MNPs or bimetallic nanoparticles composed of noble and non-precious metals are scarce and expensive, and cannot be applied on a large scale. Therefore, subsequent studies should consider affordable alternatives to MNPs or use non-precious metal particles. Secondly, based on MNPs/MOFs composites, efforts should be devoted to the development of electrochemical sensors with more sensitivity, better stability, and reproducibility. In conclusion, the nanocomposites synthesized by attaching MNPs on the surface of MOFs or encapsulating them internally have largely improved the sensing performance of electrochemical sensors and have a bright future in the sensing field.
